# In vitro comparison of pH, solubility, and radiopacity of two dual-cure resin cements

**DOI:** 10.1186/s12903-026-08103-1

**Published:** 2026-03-25

**Authors:** Evelyn Aguirre-Gil, Leonor Castro-Ramirez, José Rosas-Díaz, Enrique Yarasca-Berrocal, José Huamani-Echaccaya, Luis Cervantes-Ganoza, César Cayo-Rojas

**Affiliations:** School of Stomatology, Universidad Privada San Juan Bautista, Chorrillos and Ica, Peru

**Keywords:** Comparative study, Dental materials, Dental Cements, Dual cements

## Abstract

**Background:**

Understanding the properties of dual-cure resin cements is essential for ensuring clinical success. The present study compared the pH, solubility, and radiopacity of the dual-cure resin cements NX3 Nexus^®^ and Variolink N^®^.

**Methods:**

This in vitro experimental study prepared 30 standardized dual-cure resin cement discs (8 mm diameter × 1 mm thickness). Discs were equally allocated to two groups (*n* = 15) —NX3 Nexus^®^ and Variolink N^®^— and, within each cement, randomly assigned to three subgroups for pH, solubility, and radiopacity testing (*n* = 5 per subgroup). The non-parametric Mann–Whitney U test was employed for comparisons of solubility and pH, while the parametric Student’s t-test was employed for radiopacity. A significance level of *p* < 0.05 was considered.

**Results:**

Significant differences were observed between NX3 Nexus and Variolink N when comparing pH (*p* = 0.008) and radiopacity (*p* = 0.026). Both resin cements showed no differences when comparing solubility (*p* = 0.841).

**Conclusion:**

Under the present in vitro conditions, NX3 Nexus^®^ and Variolink N^®^ demonstrated low solubility and comparable pH. Variolink N^®^’s higher radiopacity may aid radiographic detection of excess cement and marginal assessment; thus, it may be preferred when radiographic evaluation is critical, whereas either cement is appropriate otherwise.

## Background

Dental cements create an adhesive bond between an indirect restoration and a prepared tooth, providing resistance to prosthesis dislodgement during function [1, 2]. They can be classified by chemical composition and intended use. Regardless of the material selected, viscosity and film thickness must be appropriate for cementation [3]. An optimal dental cement should be biocompatible, provide adequate working time with a short setting time, exhibit low solubility and good wear resistance, deliver high bond and compressive strengths, and be esthetically acceptable and easy to handle [4].

Resin cements are widely selected for indirect restorations owing to their esthetic qualities, favorable mechanical performance, and durable adhesion to tooth structure [5]. Historically, two curing modes were available—self-curing and light-curing—but each has limitations (e.g., incomplete conversion in deep or opaque areas for light-curing; handling and timing constraints for self-curing). These limitations led to the development of dual-cure systems, which combine a light-initiated phase with a chemical polymerization component [6, 7].

Because the effectiveness of the chemical-curing component in dual-cure resin cements can be limited—particularly over time—careful use is warranted [8]. This raises concerns about solubility in the oral environment [9] and underscores the importance of pH: acidic conditions can inhibit chemical initiation of polymerization, slow reaction kinetics, and hinder copolymerization [10]. Radiopacity is another key attribute, as it provides contrast with tooth structure and facilitates identification of excess cement, assessment of marginal adaptation, and detection of caries adjacent to restorations [9, 11]. Accordingly, understanding how dual-cure cements behave is critical to ensure durable adhesion of indirect restorations to tooth structure and long-term clinical success [12]. Thus, the physicochemical properties of these cements—pH, solubility, and radiopacity—are pivotal determinants of clinical performance and inform material selection for intracoronal and extracoronal restorations [8, 9, 12].

This in vitro study compared pH, solubility, and radiopacity of two dual-cure resin cements—NX3 Nexus^®^ and Variolink N^®^. The null hypothesis was that there would be no significant differences between the cements in pH, solubility, or radiopacity.

## Methods

### Study type and delimitation

This experimental in vitro study was conducted at the School of Stomatology of the Universidad Privada San Juan Bautista (UPSJB) and the High Technology Laboratory Certificate (ISO/IEC Standard: 17025), Lima, Peru, between February and September 2024. The study was exempted from protocol review; however, the Institutional Research Ethics Committee (CIEI) of the Universidad Privada San Juan Bautista (No. 479-2022-CIEI-UPSJB) approved its execution. This study used the CRIS Guidelines (Checklist for Reporting In-vitro Studies) [13].

### Calculation and sample selection

**Fig. 1:**
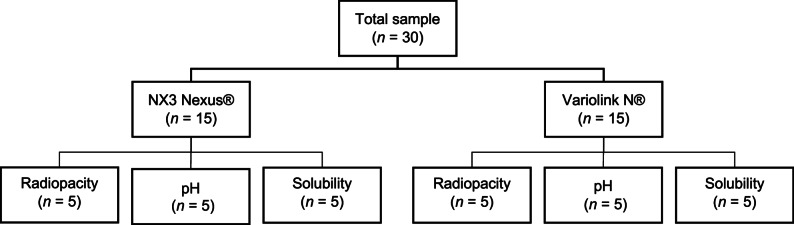
Random distribution of the groups according to the type of dual-cure resin cement and the test to be performed

A previous pilot study used 5 samples of each of the two dual-cure resin cements to determine the total sample size (*n* = 30). The samples were tested for radiopacity, pH, and solubility. The formula for the analysis of variance was applied in the statistical program G*Power version 3.1.9.7, considering a significance level (α) = 0.05, a statistical power (1-β) = 0.80, and an effect size = 10.47. Standardized dual-cure resin cement discs were prepared and equally allocated to two groups (*n* = 15 each): NX3 Nexus^®^ and Variolink N^®^. Within each cement, discs were randomly assigned to three subgroups according to the test—pH, solubility, or radiopacity—(*n* = 5 per subgroup) (Fig. 1).

### Sample characteristics and preparation

A metal mold 8 mm in diameter and 1 mm thick was used to make samples of NX3 Nexus^®^ (Kerr Corporation, Orange, USA) and Variolink N^®^ (Ivoclar Vivadent AG, Schaan, Liechtenstein) resin cements. The materials were placed in the mold, properly handled according to the manufacturer’s instructions, and the top surface was pressed with a slide plate to remove excess material [14] [Table 1]. Light activation was performed with a Bluephase N LED curing unit (Ivoclar Vivadent, Schaan, Liechtenstein) equipped with a 10-mm light-guide tip and delivering an irradiance of 1200 mW/cm². Discs (8 mm × 1 mm) were irradiated for 20 s at ~ 1 mm from the surface with the guide perpendicular to the surface; no overlaps were required because the tip diameter exceeded the disc diameter. After curing, discs were stored at 37 °C for 24 h [14].

**Table 1:** Technical profile of products used

Product	Polymerization Type	Composition	Manufacturer	Lot
NX3 Nexus	Dual-cure	HEMA, PTU, CHPO, uncured methacrylate ester monomers, titanium dioxide, and pigments	Kerr Corporation, Orange, USA	9377707
Variolink N	Dual-cure	Base: Barium glass filler and mixed oxide, dimethacrylates (BisGMA, UDMA, and TEGDMA), ytterbium trifluoride, initiators and stabilizers, pigments. Catalyst: Barium glass filler and mixed oxide, dimethacrylates, ytterbium trifluoride, initiators and stabilizers, pigments	Ivoclar Vivadent, Schaan, Liechtenstein	Z05W0M

### Measurement of radiopacity

The radiopacity test was performed according to the indications of the ISO 4049 standard [15]. A PSPIX dental imaging plate (27 × 54 mm) and a PSPIX scanner were used to obtain digital radiographic images of the samples. (Acteon Imaging, La Ciotat, France). A 10-step aluminum wedge, with thickness ranging from 1 mm to 10 millimeters, was utilized as a radiopacity reference. The imaging plate was exposed to 70 kVp, 8 mA X-Mind^®^ AC (Acteon Group, Olgiate Olona, Italy) for an exposure time of 0.125 s with an object-to-focus distance of 120 mm.

**Fig. 2:**
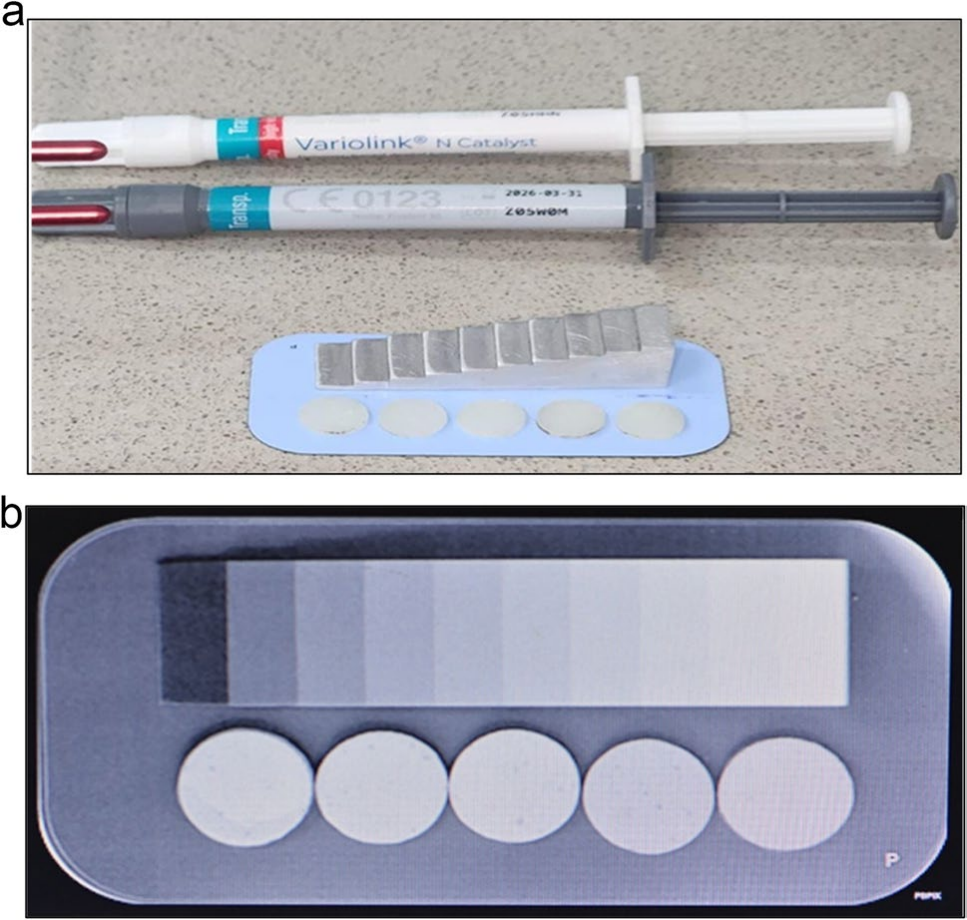
**a** Representative discs of dual-cure resin cement (NX3 Nexus^®^ and Variolink N^®^) positioned on the digital imaging plate for radiographic acquisition **b** Ten-step aluminum wedge (1–10 mm) used as the radiopacity reference standard

Images were analyzed using Adobe Photoshop CC software (Adobe System, Inc., San Jose, CA). For each sample, pixels were measured at 5 different locations [14, 16], and the average of these five measurements was calculated [14]. Sample greyscales were converted to millimeters of aluminum (mmAl) [11, 14, 16] (Fig. 2a and b).

### Measurement of pH

The dual-cure resin cements were mixed for 20 s on an acetate paper block, then spread and a PH55 digital pH meter (Milwaukee Electronics Kft, Szeged, Hungary) was attached. All samples underwent this process.

### Measurement of solubility

To ascertain the solubility of the samples, each group was stored in a desiccator (Merck KGaA, Darmstadt, Germany) and subsequently placed in an incubator at 37 °C. The samples were weighed on an analytical balance (Metter Toledo 6-digit precision weighing balance, Columbus, Ohio, USA) to the nearest 0.001 g to obtain the initial mass values (M_1_ The samples were then individually immersed in 25 mL of distilled water and incubated at 37 °C for a period of seven days. After this incubation period, each sample was dried with absorbent paper and weighed again to obtain the mass values after immersion (M_2_). The solubility was calculated using the following formula [8, 17]:

$$Sol=\frac{{M}_{1}-{M}_{2}}{V}$$

Where M_1_ is the initial mass, M_2_ is the final mass of the samples and V= volume of the sample (mm^3^).

### Statistical analysis

All the data were imported into the statistical software package SPSS, version 28.0. In order to conduct a descriptive analysis, several statistical measures were employed, including the mean, median, standard deviation, and interquartile range. To test the hypotheses, the parametric Student’s t-test for independent samples was used for radiopacity since the Shapiro-Wilk normality and Levene’s homoscedasticity statistical requirements were met. The non-parametric Mann-Whitney U test was employed for solubility and pH since the values of the two groups for both variables did not follow a normal distribution. Finally, statistical significance was set at *p* < 0.05.

## Results

Radiopacity values for NX3 Nexus^®^ and Variolink N^®^ were found to be 1.59 ± 0.30 mmAl and 2.00 ± 0.15 mmAl, respectively, when measured in grayscale. The solubility values of both resin cements were 0.46 ± 1.66 µg/mm^3^ and − 0.46 ± 0.84 µg/mm^3^, respectively. Furthermore, the pH values of both cements were 5.84 ± 0.09 and 5.32 ± 0.04, respectively. A comparison of the greyscale radiopacity (mmAl) revealed that the dual-cure resin cement Variolink N^®^ exhibited significantly greater radiopacity than the NX3 Nexus^®^ cement (*p* = 0.026). However, there was no significant difference between NX3 Nexus^®^ and Variolink N^®^ in how well they dissolved (*p* = 0.841), with average sorption rates of 1.88 µg/mm³ and 4.06 µg/mm³, respectively. It was also seen that the pH levels of NX3 Nexus^®^ were significantly higher than those of Variolink N (*p* = 0.008). **[**Table 2**].**

**Table 2:** Radiopacity (mmAl), solubility (µg/mm^3^) and hydrogen potential (pH) tests

Test	Dual-cure resin cement	*n*	Mean	SD	Median	IQR	p**	p*^,a, b^
Radiopacity	NX3 Nexus	5	1.59	0.30	1.70	0.48	0.232	0.026*^,a^
	Variolink N	5	2.00	0.15	2.00	0.21	0.324	
Solubility	NX3 Nexus	5	0.46	1.66	-1.60	2.51	0.209	0.841^b^
	Variolink N	5	-0.46	0.84	-1.51	0.58	0.007	
pH	NX3 Nexus	5	5.84	0.09	5.73	5.95	0.046	0.008*^,b^
	Variolink N	5	5.32	0.04	5.26	5.38	< 0.001	

*n *sample size*, SD *Standard Deviation*, IQR *Interquartile Range

*Significant differences (*p* < 0.05), **Shapiro Wilk normality test (*p* > 0.05, normal distribution)

^a^based on Student’s T test with homogeneous variances according to Levene test, ^b^based on Whitney Mann U test

## Discussion

This study compared pH, solubility, and radiopacity of two dual-cure resin cements—NX3 Nexus^®^ and Variolink N^®^. Variolink N^®^ exhibited higher radiopacity, whereas NX3 Nexus^®^ showed a higher pH. No differences were found for solubility, resulting in a partial rejection of the null hypothesis.

Radiopacity is critical for resin-based luting agents because it enables clinicians to detect marginal excess cement, assess marginal integrity, and identify recurrent caries adjacent to restorations [9, 11]. When radiopacity is insufficient, these defects may be missed on radiographic assessment [18]. Accordingly, it is advisable that resin cements display radiopacity comparable to or greater than enamel, to enhance clinical detectability and postoperative evaluation [19].

The results obtained indicated that Variolink N exhibited higher radiopacity than NX3 Nexus. This finding is consistent with reports by Gomes et al. [9] and Hosney et al. [16]. Several factors, including the type and chemical composition of the filler particles and their ratio, contributed to this. Resin cements consist of inorganic fillers that are dispersed in resin matrices. The primary contributors to the radiopacity of the material include elements such as barium, yttrium, ytterbium, zinc, aluminum, strontium, and zirconium [16, 18, 20]. The atomic number (Z) of the element incorporated into the material is directly correlated with its radiopacity. The higher the atomic number, the higher the X-ray absorption [20]. Thus, ytterbium, with an atomic number of 70, provides the highest radiopacity, followed by barium (Z = 56), zirconium (Z = 40), yttrium (Z = 39), strontium (Z = 38), zinc (Z = 30), titanium (Z = 22), silicon (Z = 14), and aluminum (Z = 13). The composition of Variolink N includes ytterbium trifluoride, which would be responsible for the higher radiopacity value obtained [16, 20]. Radiopacity depends on both the atomic number/density of the radiopacifier and its volume fraction within the composite matrix; in the Variolink family, high radiopacity has been associated with the use of ytterbium trifluoride (YbF_3_; Yb, Z = 70) [21]. While the exact YbF₃ proportion for Variolink N is proprietary and not publicly disclosed, product literature lists YbF₃ among its inorganic fillers. By contrast, NX3 contains barium-glass radiopaque fillers with a total inorganic filler loading reported up to ~ 70 wt% (~ 55 vol%) [22]. Therefore, differences in radiopacifier type (YbF_3_ vs. Ba-glass) and overall filler loading —rather than molecular weight alone— provide a mechanistic rationale for the greater X-ray attenuation observed in Variolink N. Another factor that may have played a role is the photosensitivity and photoreactivity of the reaction initiator in Variolink N resin cement [23–25]. It’s possible that the difference with NX3 Nexus is because Variolink N has a higher proportion of photosensitive molecules than other resin cements [25, 26]. However, the radiopacity values obtained for Variolink N (2.00 mmAl) and NX3 Nexus (1.59 mmAl) were found to be lower than those reported in several previous studies [16, 18–20]. This finding is related to several factors, such as the material composition, the parameters used for X-ray exposure, the angle at which the X-ray beam is directed, the type of sensor used, and the characteristics of the X-ray equipment employed [16, 20, 27, 28]. The spread of different inorganic charges in a resin matrix also explains the observed differences in radiopacity values [18].

Other critical properties of resin cements are their post-polymerization stability and behavior in the oral environment. However, many monomers in these materials are hydrophilic and prone to water uptake [20, 29]. Some constituents (e.g., unreacted monomers) are soluble and can migrate into water, producing weight and volume loss that is quantified as solubility [20]. In the present study, solubility did not differ significantly between NX3 Nexus^®^ and Variolink N^®^, which contrasts with the findings of Patroi et al. [30] and Mese et al. [31]. This discrepancy likely reflects methodological differences, including sample-size determination [30], performing a larger number of tests on the same disc, and the surface wear associated with repeated handling that can reduce mass [30, 32]. Although relatively high standard deviations were observed, such variability is expected in dual-cure resin cements due to hydrophilic monomers, disc-to-disc differences in degree of conversion, and edge/surface effects in small discs. According to ISO 4049:2019 [15], acceptable water solubility is ≤ 7.5 µg/mm^3^ and water sorption is ≤ 40 µg/mm^3^. In this study, both resin cements remained below these thresholds. Moreover, the occasional negative solubility values observed for Variolink N^®^ are consistent with net water sorption exceeding mass loss from leachable components (i.e., constituents that dissolve and diffuse into the storage water during immersion), as reported under ISO-aligned protocols [30, 32, 33].

In terms of pH, NX3 Nexus exhibited a higher pH value than Variolink N. Nevertheless, both materials demonstrated pH values above 3, which falls within the recommended range. Researchers have identified acidity below pH = 3 as a potential contributing factor to pulp inflammation and postoperative sensitivity in luting materials [34]. This kind of acidity may also stop the chemical activation of polymerization, which slows down the autocatalytic reaction and makes it harder for the cement to copolymerize [10].

The clinical relevance of this study lies in the fact that clinicians must consider these properties when selecting a luting material for indirect restorations such as crowns, veneers, inlays, or onlays, in which the curing light cannot fully penetrate to the adhesive interface. This highlights the need to compare two of the most commonly used dual-cure cements, as they are expected to ensure adequate polymerization in areas with limited light access. Moreover, the evaluation of physicochemical properties such as pH, solubility, and radiopacity is essential for long-term clinical success, as these factors are directly related to material stability, the risk of microleakage, and the ability to perform subsequent radiographic diagnostics [9–12].

As strengths in the present study, we can mention the use of a digital dental radiographic imaging system and a ten-step aluminum wedge as a radiopacity reference. The aluminum wedge is simple to use, accurate, and has dentin-like radiopacity. The digital dental imaging system comprises an imaging plate and a scanner, which facilitate the analysis of samples and enable the consistent production of high-quality images [35]. Furthermore, the evaluation of solubility during the first seven days was considered relevant, as it has been demonstrated that the release of residual monomers primarily occurs within this initial period following cement placement [36]. An additional strength of the study lies in the assessment of the initial pH, as it enables the analysis of the material’s initial acidity, which may influence the autocatalytic capacity of dual-cure cements [10].

The present study was unable to simulate the dynamic pH changes driven by diet and salivary composition/flow. The solubility measured in vitro may be lower than what is observed in vivo. It has been established that high salivary enzymatic activity can accelerate polymer-matrix degradation [30]. Additionally, radiographic assessment of material radiopacity may be influenced by oral fluids, overlying soft tissues, and adjacent dental structures in vivo [37].

Finally, given the limitations of the present study, it would be beneficial to evaluate longitudinal changes in solubility and pH following light activation. After initial acid neutralization and network maturation, pH is expected to rise toward neutral or mildly alkaline values, which is associated with reduced water uptake and lower hydrolytic susceptibility [38]. In addition, it would be beneficial to evaluate the degree of conversion and the mechanical properties of the materials, such as bond strength and compressive strength.

## Conclusion

Under the present in vitro conditions, NX3 Nexus^®^ and Variolink N^®^ demonstrated low solubility and comparable pH, supporting their use as luting agents. Variolink N^®^ exhibited higher radiopacity, which may facilitate radiographic detection of excess cement and assessment of marginal integrity. Thus, it may be preferred when radiographic detectability is critical (e.g., subgingival margins or implant-supported restorations), whereas either cement is appropriate otherwise. Selection may be guided by handling characteristics, adhesive protocol, and shade availability.

## Data Availability

All data analyzed during this study are available from the corresponding author on reasonable request (cesarcayorojas@gmail.com).

## Abbreviations

CIEI: Institutional Research Ethics Committee
CI: Confidence interval
CRIS: Checklist for Reporting In-vitro Studies
IQR: Interquartile Range
SD: Standard Deviation
SPSS: Statistical Package for the Social Sciences
